# Real-Time Classification of Patients with Balance Disorders vs. Normal Subjects Using a Low-Cost Small Wireless Wearable Gait Sensor

**DOI:** 10.3390/bios6040058

**Published:** 2016-11-29

**Authors:** Bhargava Teja Nukala, Taro Nakano, Amanda Rodriguez, Jerry Tsay, Jerry Lopez, Tam Q. Nguyen, Steven Zupancic, Donald Y. C. Lie

**Affiliations:** 1 The Department of Electrical & Computer Engineering, Texas Tech University (TTU), Lubbock, 79409 TX, USA; ntaro1031@gmail.com (T.N.); jerry.tsay@ttu.edu (J.T.); jerry@noisefigure.com (J.L.); tam.nguyen@ttuhsc.edu (T.Q.N.); donald.lie@ttu.edu (D.Y.C.L.); 2 (On Leave) The Department Electrical & Electronic Engineering, Tokushima University, 770-8502 Tokushima, Japan; 3 Boys Town National Research Hospital, Omaha, 68131 NE, USA; Amanda.rodriguez@boystown.org; 4 Texas Tech University Health Sciences Center (TTUHSC), Lubbock, 79439 TX, USA; steven.zupancic@ttuhsc.edu

**Keywords:** artificial neural network (ANN), back propagation (BP), binary decision trees (BDT), fall detection, fall prevention, *k*-nearest neighbors (KNN), support vector machine (SVM), wireless gait analysis sensor (WGAS)

## Abstract

Gait analysis using wearable wireless sensors can be an economical, convenient and effective way to provide diagnostic and clinical information for various health-related issues. In this work, our custom designed low-cost wireless gait analysis sensor that contains a basic inertial measurement unit (IMU) was used to collect the gait data for four patients diagnosed with balance disorders and additionally three normal subjects, each performing the Dynamic Gait Index (DGI) tests while wearing the custom wireless gait analysis sensor (WGAS). The small WGAS includes a tri-axial accelerometer integrated circuit (IC), two gyroscopes ICs and a Texas Instruments (TI) MSP430 microcontroller and is worn by each subject at the T4 position during the DGI tests. The raw gait data are wirelessly transmitted from the WGAS to a near-by PC for real-time gait data collection and analysis. In order to perform successful classification of patients vs. normal subjects, we used several different classification algorithms, such as the back propagation artificial neural network (BP-ANN), support vector machine (SVM), *k*-nearest neighbors (KNN) and binary decision trees (BDT), based on features extracted from the raw gait data of the gyroscopes and accelerometers. When the range was used as the input feature, the overall classification accuracy obtained is 100% with BP-ANN, 98% with SVM, 96% with KNN and 94% using BDT. Similar high classification accuracy results were also achieved when the standard deviation or other values were used as input features to these classifiers. These results show that gait data collected from our very low-cost wearable wireless gait sensor can effectively differentiate patients with balance disorders from normal subjects in real time using various classifiers, the success of which may eventually lead to accurate and objective diagnosis of abnormal human gaits and their underlying etiologies in the future, as more patient data are being collected.

## 1. Introduction

Analysis of human gaits has long been an active area of research, and many systems have been proposed for observing and differentiating different gait patterns and their irregularities in the literature. Many of these existing systems use appearance-based approaches by extracting features and/or positions from the images captured from different video sequences using high-speed video cameras with frame rates of 50–200 Hz. Many studies have reported that image feature extraction using biomechanical models can allow quantitative analysis of specific gait characteristics, such as joint moments and powers (i.e., kinetic analysis), joint angles, angular velocities and angular accelerations (i.e., kinematic analysis) [1]. The testing protocol of these systems includes placing the optical markers near anatomical landmarks of the body and features related to various gait patterns are extracted from video sequences. Parametric models have been used extensively to describe a set of successive image observations. A vision-based three-dimensional (3D) modeling of motions of a human subject can be achieved by using volumetric bodies (e.g., using cylinders) that represent the flesh of the human subject and a simple collection of segments with joint angles representing the skeletal structure of the human body [2]. Alternatively, 2D models that represent the projection of 3D data onto an imaging plane can also be used (i.e., 2D/3D contour modeling [3]). However, since different kinetic and kinematic methods have been developed from these sophisticated and expensive visual gait analysis systems, it can be rather challenging to directly compare the gait analysis results from different systems/methods, as there is no standardization in the visual gait analysis methodology.

In addition, the variables that can be measured during gait analysis depend on the technique and sensors selected, which makes the direct comparison of gait analysis results derived from different gait sensing systems even much more difficult. The most commonly-reported gait measurement data include the temporospatial parameters, which include walking speed, body rotations, step time, step length and the durations of the stance phase and the swing phase [4]. A basic inertial measurement unit (IMU) that includes 3D gyroscopes and accelerometers can measure angular velocity and linear acceleration for each of the X/Y/Z axes, respectively, and these inexpensive IMUs have been used as wearable sensors that provide a powerful option for human gait analysis [4,5,6]. For example, Aminian et al. [4] and Selles et al. [5] reported methods of measuring both terminal contact (TC) that defines the beginning of the swing phase, as well as the initial contact (IC) that defines the beginning of the gait cycle timing information using those body-worn sensors. On the other hand, Yoshida et al. [6] used an accelerometer/IMU sensor attached to the patient’s waist and observed frequency peaks in the anterior plane to detect leg injury. Boutaayamou et al. [7] developed a signal processing algorithm to automatically extract, on a stride-by-stride basis, four consecutive fundamental events of walking, i.e., heel strike (HS), toe strike (TS), heel-off (HO) and toe-off (TO), from wireless accelerometers applied to the right and left foot. This accelerometer-based event identification was validated in seven healthy volunteers and a total of 247 trials against reference data provided by a force plate, a kinematic 3D analysis system and a video camera. An ambulatory monitoring method using an IMU sensor for patients with Parkinson’s disease has also been developed [8,9].

**Figure 1:**
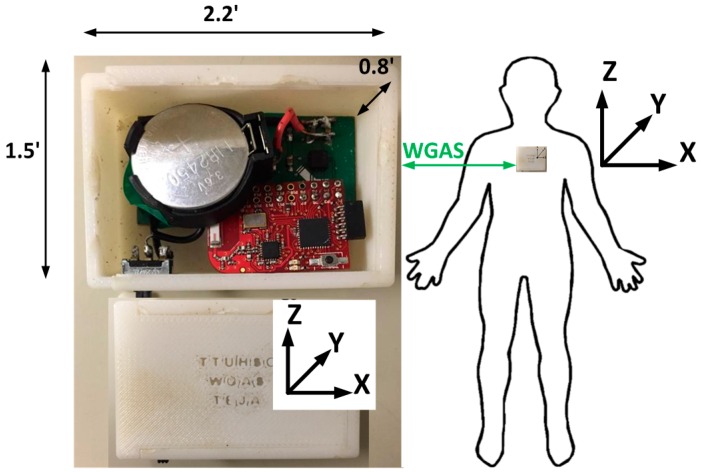
Physical structure and dimension of our custom-designed wireless gait analysis sensor (WGAS) hardware and the orientation placed during tests at T4 (back).

We have, therefore, designed a custom low-cost wireless gait analysis sensor (WGAS), which can be used for both fall detection and gait analysis. We reported to have demonstrated measured fall detection rates of 99% in classification accuracies among young volunteers using a similar WGAS with the BP-ANN and SVM classification algorithms [10,11,12]. This paper, however, studies and reports our new WGAS that is used specifically for gait analysis to detect patients with balance disorders among normal subjects while using various classification algorithms and input features to check the speed and accuracy of each classifier. This WGAS is placed and tested at the T4 position on the back of each subject, as shown in Figure 1. For the learning of the classification algorithms, we will first use the following six input features for the X/Y/Z axes extracted from the raw data taken from the WGAS (i.e., *R_ω_*: range of angular velocity; *R_A_*: range of acceleration, as shown in Equations (1) and (2)). They are used as one of the input features sets for these classifiers that yield excellent classification accuracies, and the details of the WGAS and our experimentation and analysis will be explained next.

(1) $$R_{\omega ,x}=\max(\omega_{x})-\min(\omega_{x}),R_{\omega ,y}=\max(\omega_{y})-\min(\omega_{y}),R_{\omega ,z}=\max(\omega_{z})-\min(\omega_{z})$$

(2) $$R_{A,x}=\max(A_{x})-\min(A_{x}),R_{A,y}=\max(A_{y})-\min(A_{y}),R_{A,z}=\max(A_{z})-\min(A_{z})$$

### Wireless Gait Analysis Sensor

**Figure 2:**
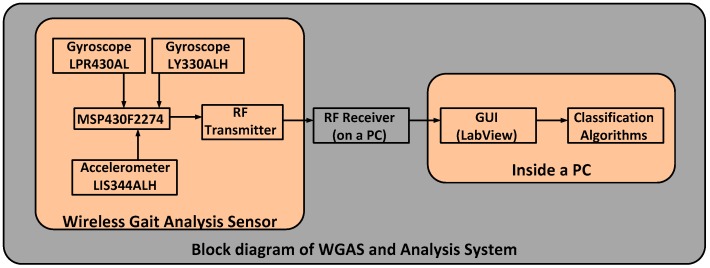
A simplified block diagram of the gait analysis/classification system using our WGAS.

The custom-designed WGAS consists of a three-axis linear accelerometer IC, a single-axis gyroscope IC and a dual-axis gyroscope IC to measure 3D human body translations and rotations during a gait pattern; these ICs are designed with the help of micro-electrical and mechanical system (MEMS) sensors. Our WGAS measures the linear acceleration and angular rotations of the body movements, and there is no need for a magnetometer for our application of gait analysis and gait classification. Furthermore, the sensor has a dual- and single-axis gyroscope instead of a tri-axial gyroscope, because the particular analog MEMS tri-axial gyroscope was not available on the market during the design of the WGAS, but it is now. The future design of our WGAS can have a more compact IMU with a tri-axial single accelerometer IC and a single-gyroscope IC. However, we do not believe this will affect the sensing results whether one IC or two ICs are used. The details of these ICs with their manufacturers’ information can be found in [10,11]. This WGAS system is supported by a Texas Instruments (TI) MSP430 microcontroller (Texas Instruments, Dallas, TA, USA) and a wireless 2.4 GHz USB transceiver using the TI SimpliciTI™ protocol (Texas Instruments, Dallas, TA, USA) with a wireless communication range of ~12 meters (40 ft). An overall simplified system block diagram for the WGAS analysis system is shown in Figure 2.

**Figure 3:**
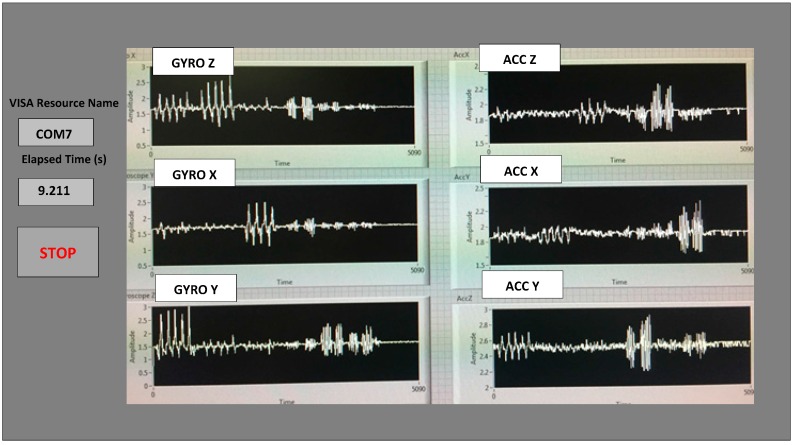
An example output on the LABVIEW GUI of the data wirelessly transmitted from the WGAS to a nearby PC, showing the real-time data for the six monitored signals. For example, the “GYRO X” signal is the angular velocity measured from the gyroscope centered on the X axis, and the “ACC X” signal is the acceleration measured from the accelerometer along the X axis, respectively.

The two AAA batteries used in our earlier wired sensor [13] were replaced by a single rechargeable Li-ion coin battery, providing a battery lifetime of ~40 h of continuous operation time with each recharge. The PCB, coin battery and the microcontroller are placed in a specially-designed 3D-printed plastic box (2.2′ × 1.5′ × 0.8′) with a total weight of 42 g. The design of the box was done with the 3D modeling software Rhinoceros (Rhino, Robert McNeel & Associates, WA, USA) and printed using a 3D printer with acrylonitrile butadiene styrene (ABS) plastic. The box has a sliding lid and is shown in Figure 1. The accelerometer data are sampled at 160 Hz and digitized to eight bits, with its output scaled to ±6 g at Δ V = ±6 g/VDD (VDD, Supply voltage = 3.6 V) for each axis. The gyroscope data are also sampled at 160 Hz and digitized to eight bits, with its output scaled to 300°/s (dps) at ΔV = ±300 dps. Multiplied by So (note the typical So (sensitivity) value is 3.752 mV/dps for the accelerometer and 3.33 mV/dps for the gyroscopes). The sensor orientation and position on the body during testing are also shown in Figure 1. The sensor is carefully secured to the subjects during testing to avoid artifacts. A cloth is used to secure the WGAS by tying it tightly at the T4 position of the patient’s back. The cloth is also attached to the shirt of the subject by using plastic tape. In addition, Velcro is attached to the sensor box that firmly attaches to the shirt, as well, to make sure that the sensor does not move around during the DGI tests shown in Table 1. The method described above proved that it significantly reduced the outliers in the measured datasets from the sensor. The microcontroller and the transceiver unit enable the real-time wireless transmission of the six-dimensional gait data to the nearby PC, where a LABVIEW™ program is used for designing the graphical user interface (GUI), as shown in Figure 3. The DC values for the six signals are not the same as the battery charges and discharges, and the calibration to make them the same DC level was not done to simplify the WGAS design, as we have ascertained that the detected AC signals from WGAS are really the ones that contain the most useful gait information, as will be shown in the remaining of this paper.

## 2. Experimental Section

All four patients and the three normal subjects performed the 7 Dynamic Gait Index (DGI) tests [10,14] with the details shown in Table 1. Therefore, a total of 28 dynamic gait tests were performed on 4 patients and 21 tests on three normal subjects. During all of the tests, the WGAS is placed at the T4 position (at back) for all of the subjects involved. Our WGAS sensor has been accepted by the Texas Tech University Health Sciences Center (TTUHSC) Internal Review Board (IRB) with study title “Fall risk identification and assessment using body worn sensors (CRI12-030 Fall study)”.

**Figure 4:**
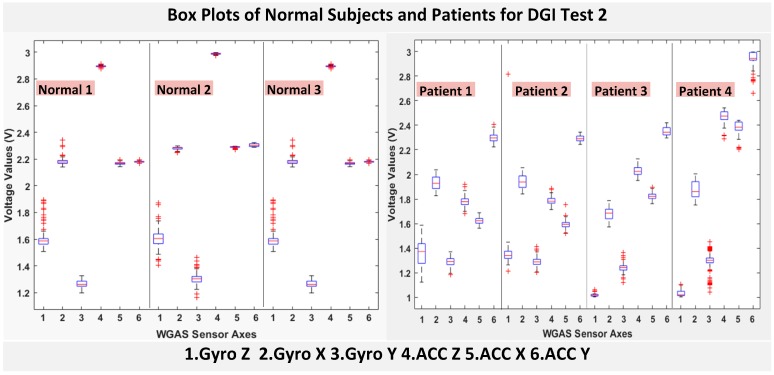
Box plots of DGI Test 2: Data for normal subjects (**left**) vs. patients (**right**).

**Figure 5:**
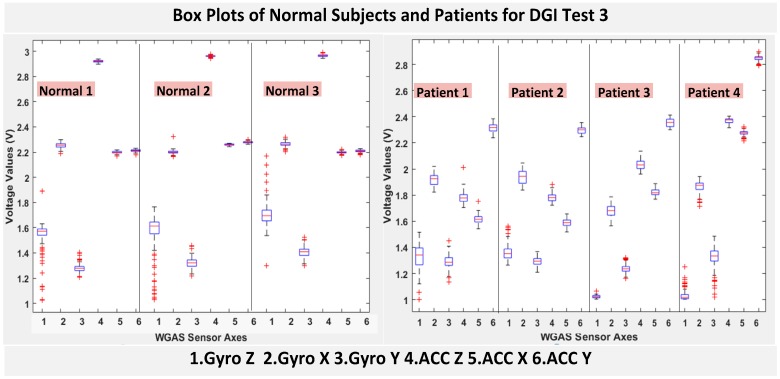
Box plots of DGI Test 3: Data for normal subjects (**left**) vs. patients (**right**).

**Figure 6:**
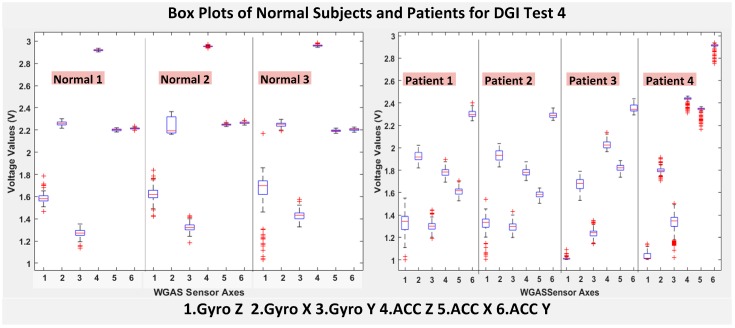
Box plots of DGI Test 4: Data for normal subjects (**left**) vs. patients (**right**).

**Figure 7:**
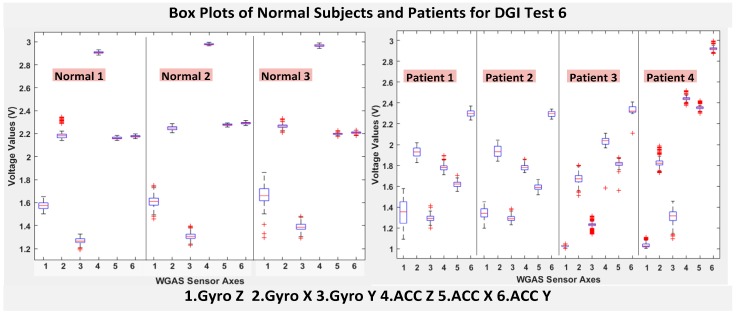
Box plots of DGI Test 6: Data for normal subjects (**left**) vs. patients (**right**).

**Figure 8:**
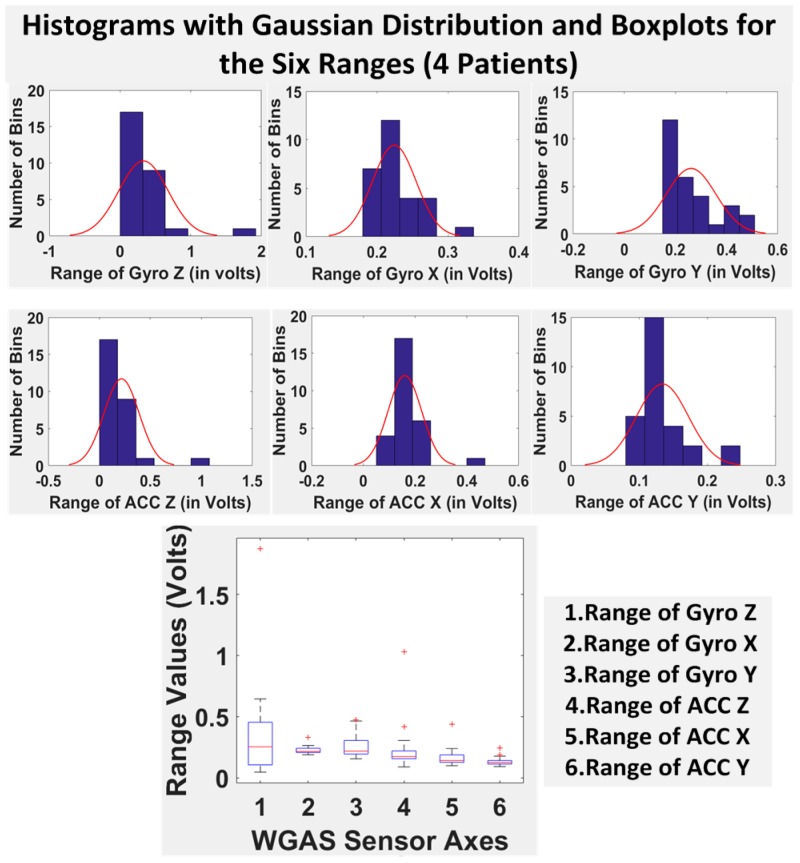
Histograms with the Gaussian distribution and box plots for the six ranges combining all of the DGI tests from the four patients.

**Figure 9:**
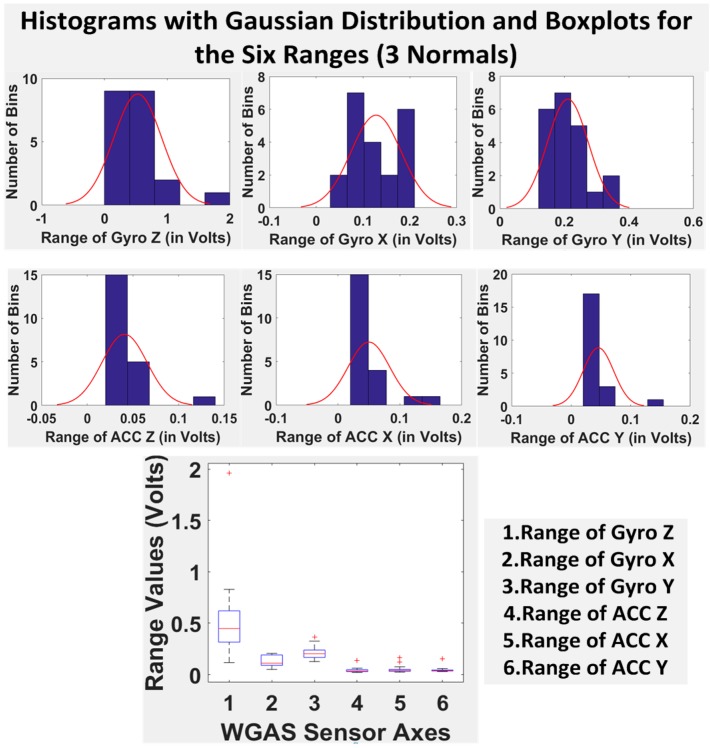
Histograms with the Gaussian distribution and box plots for the six ranges combining all of the DGI tests from the three normal subjects.

The six features of the raw data on range from the gyroscopes and the accelerometers from all of the testing subjects are important, and they form the inputs for training the classification algorithms. These include the range of angular velocity in the X/Y/Z direction (i.e., *R_ωx_*, from “GYRO X”; *R_ωy_*, from “GYRO Y”; and *R_ωz_*, from “GYRO Z”) and the range of acceleration in the X/Y/Z direction (i.e., *R_Ax_*_,_ from “ACC X”; *R_Ay_*, from “ACC Y”; and *R_Az_*, from “ACC Z”). The data were obtained using a 1.7 GHz PC with 4 GB of RAM, Windows 8 OS, and using MATLAB R2015b. We compared the box plots of the four patients and three normal subjects to show several key test data statistics: the median, mean, range (highest to lowest values) and interquartile range (IQR) of DGI Tests 2, 3, 4 and 6, as shown in Figure 4, Figure 5, Figure 6 and Figure 7. These four DGI tests are selected and plotted here; they can show better visual comparisons than the other three DGI tests; some detailed statistics of all DGI tests are also listed later in this paper i.e., at the end of Section 2 (e.g., Figure 8 and Figure 9, etc.)

Moreover, the average of STDEV (i.e., standard deviation), Range, Mean, Median, and IQR of DGI Tests 2 and 7 are calculated and shown in Table 2 and Table 3.

As shown in Figure 4, the box plots of the patients’ data appear to be mostly larger than those of the normal subjects for DGI Test 2 for all extracted features, and they look significantly tighter for all of the 3-axial acceleration data for all DGI tests as shown in Figure 4, Figure 5, Figure 6 and Figure 7 (i.e., “ACC X”, “ACC Y” and “ACC Z”). The tighter the box plot distributions suggest the normal subjects are walking more steadily with less wobbling or sways than patients during the DGI tests.

Besides the larger box plots associated with larger STDEV and IQR for patients, in all box plots, we have also observed the median/mean values of the acceleration measured on the X and Z axes (i.e., “ACC X”, “ACC Z”) of the normal subjects are significantly larger from those of patients for all of the DGI tests, and we have shown this difference in Table 2 and Table 3 for DGI Test 2 and Test 7 as examples for better illustration. This contrast might be explained because walking gaits of normal subjects are considerably different from those of patients, where they walk freely with more accelerations along the X/Z direction from their center of mass (CoM), while patients of balance disorders typically walk with a decrease of speed, shortened stride length and other associated factors [4,5]. It is also interesting to notice that the median/mean values of ACC Y of the normal subjects are not so different from those of patients. This might be due to that during the DGI tests, the Y axis is parallel to the walking direction, while the X and Z axes are perpendicular to the walking direction, and therefore, we did not see much change in the median/mean values along the Y axis. We will need to look at these effects related to the median/mean values from accelerometers closely after more patient data can be collected to hopefully understand them better.

Moreover, as mentioned before, when the gait data’s STDEV is smaller, one would expect the walking to be more steady or stable. We can indeed see in the box plots and tables that the normal subjects’ STDEV values on Gyro X and Y and ACC X, Y and Z are all smaller than those of patients. However, the normal subjects’ STDEV data of Gyro Z are actually than that of the patients, suggesting that normal subjects may rotate their bodies around the Z axis naturally more and with more variation than patients of balance disorders; their faster walking speed than the patients may contribute to this effect, as well. Note we would really need to collect more patient gait data to improve the statistics and analysis details especially on the gyroscope data, as we can see that for all seven DGI tests, the STDEVs of Gyro Z data for Patients No. 1 and No. 2 are so different from those of Patients No. 3 and No. 4 (see Figure 4, Figure 5, Figure 6 and Figure 7). To see this better, we have also shown the values of the STDEV for normal subjects and patients for DGI Test 2 in Table 4. Therefore, one can see from Table 4 that the STDEVs of Gyro Z for normal subjects are actually slightly lower than those of “Patient 1” and “Patient 2”, but much greater than those of “patient “and “Patient 4”; therefore, the average STDEV of Gyro Z among normal subjects becomes larger than that of patients.

Finally, not surprisingly, Table 3 shows that the average range values of Gyro X, Y, Z and also ACC X, Y, Z for the patients’ gait data are all greater than those of the normal subjects’ for all of the DGI tests, except for the range of the Gyro Z data of the DGI Test 7, probably for the reasons stated before that the normal subjects may rotate their bodies around the Z axis naturally more with faster walking speeds and, therefore, with more variation and range difference than patients of balance disorders. Moreover, Figure 4, Figure 5, Figure 6 and Figure 7 show the box plots of DGI Tests 2, 3, 4 and 6 that group normal subjects as one group vs. patients as another group. From these points, the DGI Tests 2, 3, 4 and 6 present as better tests than the DGI Tests 1, 5 and 7 for differentiating patients from normal subjects. Having checked those basic data statistics, we have decided to first use the range values of the patients and normal subjects from all six sensor axes and for all 7 DGI tests taken as the single input features for the classification algorithms. We will later also use STDEV, etc., as input features to these classifiers and compare them, as well.

Finally, we have shown in Table 5 the STDEV, range, median, mean and IQR for normal subjects vs. patients for all seven DGI tests. To see the distributions on range better, the histograms of the data with normal distribution are now also shown for each of the six features for both patients and normal subjects in Figure 8 and Figure 9. The box plots of normal subjects and patients are also shown for these six features. It can be clearly seen that the features related to the acceleration data (i.e., the “ACC” data) look tighter in the distribution for the normal subjects than for the patients, especially for ACC X and ACC Z. After checking the raw gait data carefully to ensure the data integrity, we are now ready to show the classification algorithms and classification results next.

### 2.1. Classification Algorithms

Classification algorithms were used on the 6 features extracted from all 7 DGI tests and for all testing subjects to differentiate patients vs. normal subjects from all of the data collected.

#### 2.1.1. Back Propagation Artificial Neural Network 

**Figure 10:**
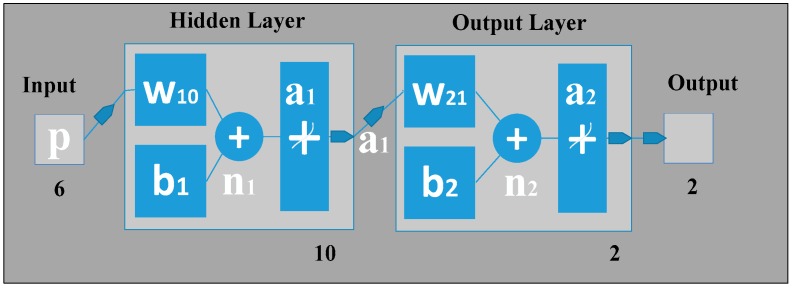
The 3-layer back propagation artificial neural network (BP-ANN) topology used for this work.

An artificial neural network (ANN) can be seen as a machine that is designed to mimic how the brain performs a particular task or function of interest. Using ANN as a classifier has several advantages, such as: (1) neural networks are data-driven self-adaptive methods that can adjust themselves to the input data without using explicit mathematical functions; (2) it is a nonlinear model that can model most real-world problems; (3) neural networks are able to estimate Bayesian posterior probability, which can provide the basic estimation of classification rules [10]. To train the feed-forward ANN classifier in this work, back propagation (BP) was applied on the input features with scaled conjugate gradient (SCG) learning [10,11]. Similar to the neural network [10], our gait classification neural network also has three layers in which the input layer has six neurons that correspond to the six input feature values. The hidden layer can automatically extract the features of the input patterns. There is no definite rule used to determine the number of neurons in the hidden layer; it is a hit-and-trial method. Here, in our classification study using ANN, there is one hidden layer holding 10 hidden neurons, the number of which was optimized by adjusting the size of hidden neurons (from 1 to 15) as shown in Figure 10. For the hidden layer, a hyperbolic tangent sigmoid transfer function is used for each neuron. At the hidden layer, it is used to calculate network output from its input. The tangent hyperbolic function and its fast approximation are given by the following equation:

(3) $$a_{i1}=tansig\left(n_{i1}\right)=\frac{2}{\exp\left(-2n_{i1}\right)+1}-1$$

where $a_{i1}$ is the *i*-th element of the $a_{1}$ vector containing the output from the hidden neurons, $n_{i1}$ is the *i*-th element of $n_{1}$ vector containing the net input going into the hidden units and $n_{1}$ is calculated by using the formula:

(4) $$n_{1}=w_{10}p+b_{1}$$

where *p* is the input pattern, $b_{1}$ is the bias vector and $w_{10}$ is the weight matrix between the hidden layer and the output layer.

The output layer is designed based on the required output of the neural network. Here, we have used two output neurons corresponding to the two target classes the network needs to differentiate (i.e., the features of all patients are considered as Class 1, and all features of normal subjects are as Class 2). The pure linear activation function is selected for the output, given by the following equation:

(5) $$a_{2}=n_{2}$$

where $a_{2}$ is the column vector coming from the output layer and $n_{2}$ is the output net inputs going into the output layer, which can be calculated by using the following formula:

(6) $$n_{2}=w_{21}a_{1}+b_{2}$$

Note here that *b_2_* is the bias at the second layer; $w_{21}$ is the synaptic weights at the hidden layer and output layer; and *a_1_* is the column vector containing the outputs from the hidden layer.

Here, we have used the back propagation learning algorithm that consists of two paths; the forward path and the backward path. The forward path includes creating the feed-forward network, initializing weights, simulation and training the network. The network weights and biases are updated in the backward path. Here, we have used the scaled conjugate gradient (SCG) training algorithm that uses the gradient of the performance function to determine how to adjust weights to minimize the performance function. An iteration of this algorithm can be written as:

(7) $$\;x_{k+1}=x_{k}-\alpha_{k}g_{k}$$

where $x_{k}$ is the vector of current weights and biases, $g_{k}$ is the current gradient and $\alpha_{k}$ is the learning rate.

#### 2.1.2. Support Vector Machine 

The support vector machine (SVM) method is popular for performing pattern recognition/classification on two categories of data with supervised learning. In our work, SVM was implemented similar to [10,15,16] to classify patients from normal subjects using the gait data from our WGAS system. A linear kernel was used for training the SVM classifier, which finds the maximum-margin hyper-plane from the given training dataset D, and it can be described as:

(8) $$D={\left\{\left({\vec{x}}_{i},y_{i}\right)|{\vec{x}}_{i}\in i^{p},y_{i}\in \left\{-1,1\right\}\right\}}_{i=1}^{n}$$

where $y_{i}$ is either 1 or −1, and *n* is the number of training data. Each ${\vec{x}}_{i}$ is a p-dimensional vector having the feature quantity *R*. Any hyper-plane can be written as:

(9) $$\vec{w}\cdot \vec{x}-b=0$$

where $\vec{w}$ is the vector to the hyper-plane. If the training data are linearly separable, the hyper-plane can be described as [15]:

(10) $$\vec{w}\cdot \vec{x}-b=1\;\mathrm{and}\;\vec{w}\cdot \vec{x}-b=-1$$

The distance between these two hyper-planes is $2/∥\vec{w}∥$, so the purpose is to minimize $∥\vec{w}∥$.

In general, it is hard to separate the training data linearly. When the training data are not linearly separable, the hyper-plane can be described as:

(11) $$Min\;\frac{1}{2}{∥\vec{w}∥}^{2}+C\sum_{i=1}^{l}\varepsilon_{i}$$

where parameter C determines a trade-off between the error on the training set and the separation of the two classes. Here, ε is a set of slack variables. The dual problems lie in maximizing the following function with respect to the Lagrange multiplier α [15]:

(12) $$Max\;\overset{l}{\underset{i=1}{\sum }}\alpha_{i}-\;\frac{1}{2}\overset{l}{\underset{j=1}{\sum }}\alpha_{i}\alpha_{j}y_{i}y_{j}(x_{i}\cdot x_{j})$$

subject to $0\;\leq \;\alpha_{i}\;\leq C\;\left(i=1,\ldots ,l\right)$:

(13) $$\overset{l}{\underset{i=0}{\sum }}\alpha_{i}y_{i}=0$$

#### 2.1.3. *K*-Nearest Neighbor 

The *K-*nearest neighbors algorithm (KNN) is a simple, efficient non-parametric method used for classification and regression in pattern recognition, object recognition, etc. [17]. In both cases, the input consists of the *K* closest training examples in the feature space.

In KNN classification, the object or an unknown sample is classified by assigning to a test pattern the class label of its *K* nearest neighbors. The object is classified based on the category of its nearest neighbors through a voting procedure. Majority votes of its neighbors are considered during classification, with the object assigned to the most common class among its *K* nearest neighbors (*K* is a positive integer, typically small). If *K* = 1, then there is only one nearest neighbor, and the object is simply assigned to that class. Because of its simplicity and efficiency, the KNN*-*based algorithms are widely used in many applications.

#### 2.1.4. Binary Decision Tree 

Binary decision tree (BDT), also called as decision trees or classification trees, can predict responses to data as a classifier. To predict a response, one needs to follow the decisions in the tree from the root (beginning) node down to a leaf node, where the leaf node contains the response. BDT give responses that are nominal, such as “true” or “false”. Here, we have used BDT for the patients’ and normal subjects’ gait data classification. In data mining, BDT can also be used for regression and generalization of a given set of data [18]. Data come in records of the form:

(14) $$\left(x,Y\right)=\left(x_{1},x_{2},x_{3},\ldots \ldots ,x_{k},Y\right)$$

The dependent variable in Equation (14), *Y*, is the target variable that we are trying to predict, classify or generalize. The vector *x* consists of the input variables, *x*_1_, *x*_2_, *x*_3_, etc., that are used for classification.

**Figure 11:**
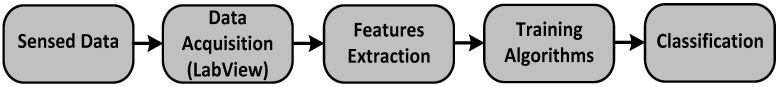
Overall data acquisition, feature extraction and data classification analysis flow for the gait analysis system using our WGAS.

The overall system flow for our real-time gait classification system is shown in Figure 11. We will now present the classification results from our WGAS system using these aforementioned classifiers next.

## 3. Results and Discussion

**Figure 12:**
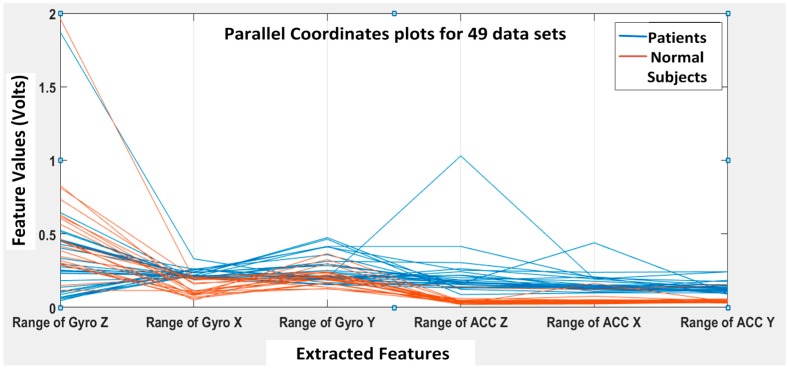
Six extracted features from the WGAS data collected on range (i.e., the difference between maximum and minimum) for four patients and three normal subjects. A total of seven persons each had all seven DGI tests done, so there are 7 × 7 = 49 cases.

The training and testing datasets were divided into 70:30 in percentages for the BP-ANN algorithm. The rest of the three algorithms used the *K*-fold cross validation method with *K* = 6 [19]. The *K*-fold cross validation method generalizes the approach by segmenting the data into *K* equally-sized partitions. During each run, one of the partitions is chosen for testing, while the rest of them are used for training. The procedure is repeated *K* times so that each partition is used for testing exactly once. Again, the total error is found by summing up the errors for *K* runs. A parallel coordinate plot is shown in Figure 12, illustrating the six features for all 49 datasets (seven subjects with seven DGI tests). The blue lines refer to extracted features for patients, and the brown lines refer to the extracted features for normal subjects.

From Figure 12, we can clearly see that the features F4, F5 and F6 of the normal subjects (i.e., brown lines; range of ACC Z, range of ACC X and range of ACC Y, respectively) are almost concentrated at 0.1 Volts in the feature space, but the F4, F5 and F6 of the patients (i.e., blue lines) are varying from 0.2 to 1 in the feature space. Therefore, the linear acceleration features (i.e., F4, F5 and F6) from the accelerometer data can be used to differentiate patients from normal subjects. However, the gyroscopic features (i.e., F1, F2 and F3) vary rather differently, and they appear not that different for normal subjects vs. patients. Nevertheless, we found that the gyroscopic features are still important for the classification algorithms to possibly increase their classification accuracy.

**Figure 13:**
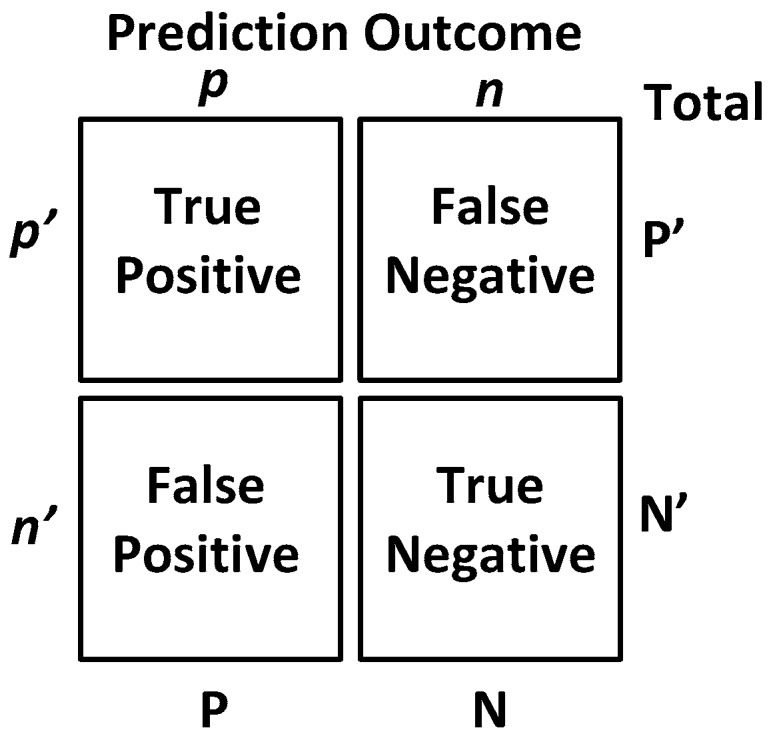
The definition of the confusion matrix [20], which is a tool for analyzing how well a classifier can recognize input features of different classes by tabulating the classification results correctly/incorrectly as predicted by the classification model.

Before we show the classification results, we would like to introduce some straight-forward graphical representation and figures-of-merit used to evaluate a classifier. For example, a “confusion matrix” is a useful and simple tool for analyzing/representing how well a classifier can recognize input features of different classes [20]. It tabulates the outcomes performed by a classifier for both correctly- and incorrectly-classified cases. Its definition is shown in Figure 13.

Additionally, popular performance metrics for biosensors, such as sensitivity, specificity, positive predictive value (PPV) or precision, F-measure and accuracy, are also used in this work as defined below [21], where TP = true positives, TN = true negatives, FP = false positives, FN = false negatives.

Sensitivity (recall): This measures the actual members of the class that are correctly identified as such. It is also referred to as the true positive rate (TPR). It is defined as the fraction of positive examples predicted correctly by the classification model.

(15) $$\mathrm{Sensitivity}=\frac{\mathrm{TP}}{\mathrm{TP}+\mathrm{FN}}$$

Classifiers with large sensitivity have very few positive examples misclassified as the negative class.

Specificity: This is also known as the true negative rate. It is defined as the fraction of total negative examples that are predicted correctly by the model/classifier.

(16) $$\mathrm{Specificity}=\;\frac{\mathrm{TN}}{\mathrm{TN}+\mathrm{FP}}$$

Precision (positive predictive value): Precision determines the fraction of records that actually turns out to be positive in the group the classifier has declared as positive class.

(17) $$\mathrm{Precision}=\frac{\mathrm{TP}}{\mathrm{TP}+\mathrm{FP}}$$

The higher the precision is, the lower the number of false positive errors committed by the classifier.

Negative predictive value (NPV): This is the proportion of samples that do not belong to the class under consideration and that are correctly identified as non-members of the class.

(18) $$\mathrm{NPV}=\frac{\mathrm{TN}}{\mathrm{TN}+\mathrm{FN}}$$

F − measure: Precision and sensitivity are two widely-used metrics for evaluating the correctness of a classifier or a pattern recognition algorithm. Building a model that maximizes both precision and sensitivity is the key challenge for classification algorithms. Precision and sensitivity can be summarized into another metric known as the F-measure, which is the harmonic mean of precision and sensitivity, given by,

(19) $$F-\mathrm{measure}=\frac{2\times \mathrm{Precision}\times \mathrm{Sensitivity}}{\mathrm{Precision}+\mathrm{Sensitivity}}$$

Accuracy: Accuracy is used as a statistical measure of how well a binary classification test identifies or excludes a condition. It is a measure of the proportion of the true results as defined by Equation (20):

(20) $$\mathrm{Accuracy}=\frac{\mathrm{TP}+\mathrm{TN}}{\mathrm{TP}+\mathrm{FP}+\mathrm{TN}+\mathrm{FN}}$$

Now, we are ready to discuss the classification results. Considering the importance of all of these six features from the range data, each classifier was trained with the inputs of all six features. The back propagation artificial neural network (BP-ANN) achieved an impressive 100% accuracy with the SCG learning. This SCG algorithm performs the search and chooses the step size by using the information from the second order error function from the neural network. The SCG training is optimized by the parameter sigma σ (which determines the change in weight for the second derivative approximation) and lambda λ (which regulates the indefiniteness of the Hessian). The values of σ and λ were taken as $5\cdot e^{-5}$ and $5\cdot e^{-7}$, respectively.

**Figure 14:**
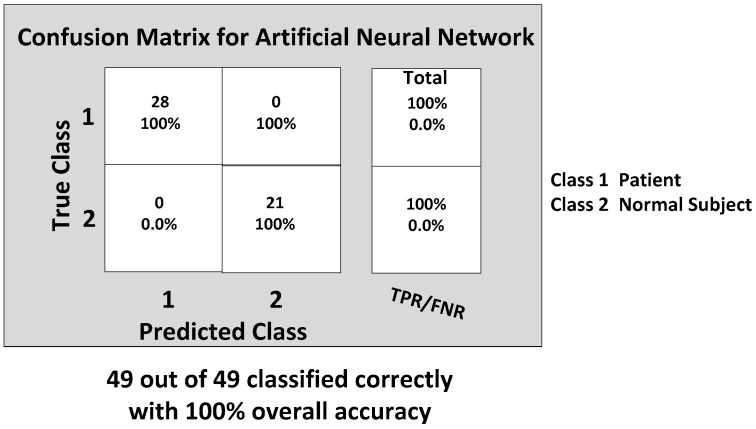
The final confusion matrix showing the gait classification results using the BP-ANN classification model with the true positive rate (TPR) and false positive rate (FPR).

The confusion matrix of the BP-ANN classifier is shown in Figure 14, which gives the TP, TN, FP and FN values.

**Figure 15:**
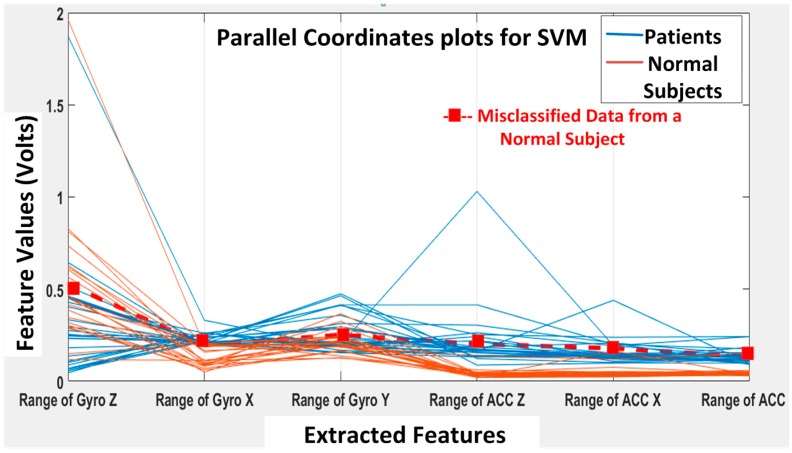
SVM classification results: misclassification shown in the feature space with only one case misclassified (i.e., DGI Test 7 of the first normal subject is classified as the “patient” class).

**Figure 16:**
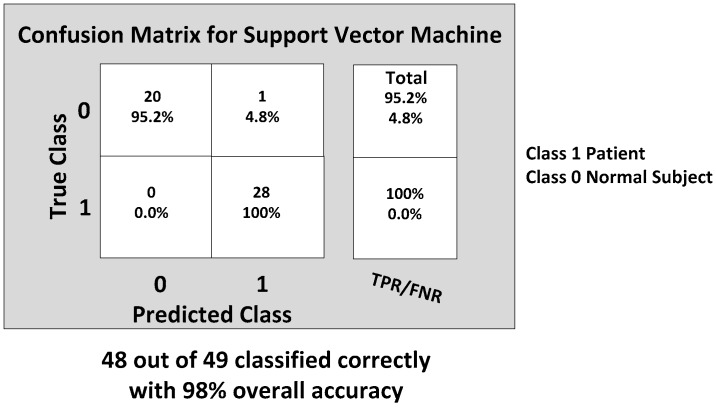
The final confusion matrix showing the gait classification results using the SVM classification model with the true positive rate (TPR) and false positive rate (FPR).

Next, the SVM classifier with a linear kernel achieved 98% overall accuracy with only one misclassification from the 49 feature datasets. The misclassified data are shown in Figure 15, and the corresponding confusion matrix is shown in Figure 16. The only one misclassification is Case 7 of the first normal subject, which was misclassified as the patient class.

**Figure 17:**
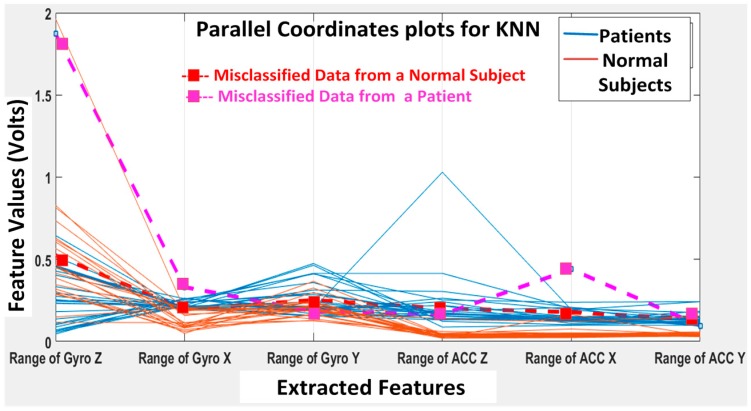
*K*-nearest neighbor (KNN) classification results: two misclassifications shown in the feature space: Case 7 (DGI 7) of the first normal subject and Case 1 (DGI 1) of the second patient classified as patient and normal subject, respectively.

**Figure 18:**
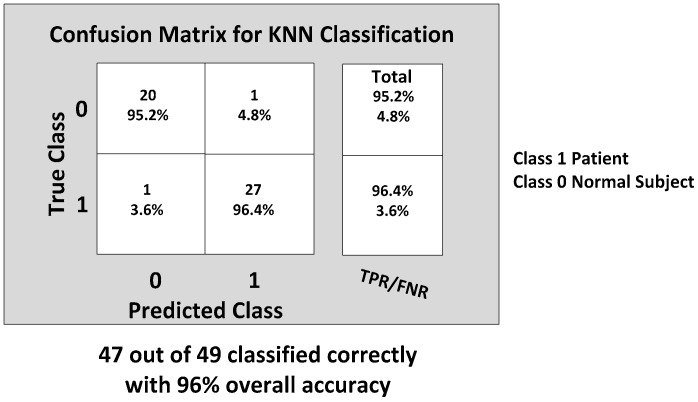
The final Confusion matrix showing the gait classification results using the KNN classification model with the TPR and False Positive Rate (FPR).

**Figure 19:**
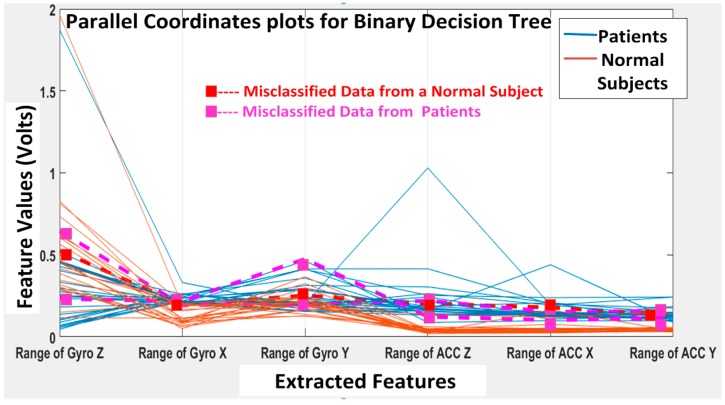
Binary decision tree (BDT) classification results: three misclassifications shown in the feature space: Case 7 (DGI 7) of the first normal subject, Case 4 (DGI 4) of the fourth patient and Case 6 (DGI 6) of the first patient.

**Figure 20:**
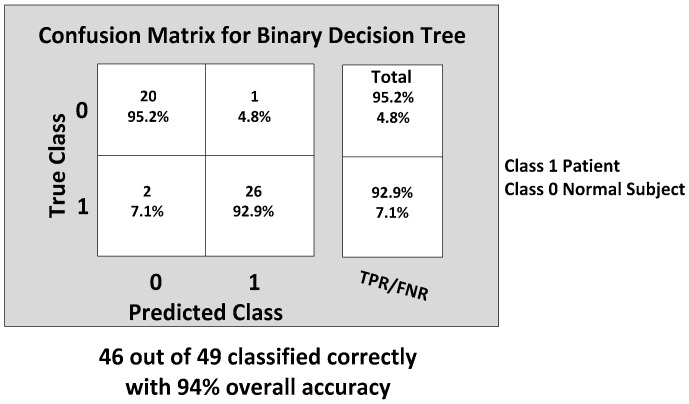
Confusion matrix showing the gait classification results using the binary decision tree (BDT) classification model with the true positive rate (TPR) and false positive rate (FPR).

The KNN and BDT algorithms were also trained on the data collected and achieved 96% and 94% in accuracy, respectively. The parallel coordinates plots and the confusion matrices are shown in Figure 17, Figure 18, Figure 19 and Figure 20, respectively. For the KNN classifier, there are two misclassifications: Case 7 of the first normal subject and Case 1 of the second patient were misclassified. Somewhat similarly, for the BDT algorithm, there are three misclassifications: Case 7 of the first normal subject, Case 4 of the fourth patient and Case 6 of the first patient.

From the classification results of the trained algorithms, we can clearly see that our WGAS system is robust enough to differentiate patients and the normal subjects with the simple features extracted from the raw accelerometer and gyroscope data as each classifier exhibits 94%–100% high accuracy. The comparison and the performance of the algorithms are shown in the Table 6 as below, where the range data have been used as the input features to the classifiers.

Next, we also tried different statistical parameters to use as the input features to check the impact on classifier accuracy. When only STDEV is used as the input feature, Table 7 suggests the classification accuracies for all classifiers degraded, while SVM classification accuracy is now 94%, slightly better than the other classifiers. When both range and STDEV are used as the input features, the SVM’s accuracy improves to 98%, and all classifiers have higher than 94% accuracy. The best gait classification accuracy still occurs when only range data are used as input features; in that case, the BP-ANN outperformed all other classifiers with 100% accuracy.

Finally, we need to compare the running time of each of the classifiers to see which algorithm is the fastest to perform this gait classification in real time; we also compared the speed when different input features are used to train the classification algorithms, and the results are shown in Table 8. It is apparent that a simple, but very fast BP-ANN classifier appears to be the best classifier to differentiate patients of balance disorders vs. the normal subjects in real time. SVM also has achieved 100% precision and specificity, but 98% accuracy; however, it is significantly slower than BP-ANN, KNN or BDT. We are collecting more gait data from patients and normal subjects now to improve the data statistics and to ascertain if BP-ANN and SVM would still be the best algorithms for real-time patient classification.

Table 9 shows the results of this work compared with previously-published work in the literature. The Mannini et al. [22] used three IMUs featuring a tri-axial accelerometer and a tri-axial gyroscope and collected the raw data from the testing subjects. They have achieved 90.5% classification accuracy using the RBF (radial basis function) kernel SVM classification algorithm by including time domain features, like the mean value, STDEV, maximum, minimum and range in their feature extraction. The Tahir et al. [23] used both ANN and SVM classifier algorithms for the gait classification in Parkinson’s disease patients. They have used the SVM classifier with the RBF kernel in distinguishing normal and patients based on kinetic features. They have also used ANN with the Levenberg–Marquardt training algorithm and achieved 98.2% and 96.9% classification accuracy, respectively.

The Bregg et al. [24] applied an ANN and SVM for the automatic recognition of young-old gait types from their respective gait patterns. Minimum foot clearance (MFC) data of young and elderly participants were analyzed using a PEAK-2D motion analysis system during a 20-min continuous walk on a treadmill at a self-selected walking speed. Gait features extracted from Poincaré plot images were used to train the SVM and ANN. Cross-validation test results indicate that their generalization performance of the SVM was on average 83.3% (±2.9) to recognize young and elderly gait patterns, compared to a neural network’s accuracy of 75.0% (±5.0). The same research group of [24] used a synchronized PEAK 3D motion analysis system and a force platform during normal walking for young and elderly subjects and achieved 83.3% vs. 91.7% generalization performance for ANN and SVM, respectively, as reported in [25]. The Hasin et al. [26] used both SVM and ANN for gait recognition by extracting geometry and texture features from the frame sequence of the video when the person is walking. They have used polynomial SVM of order three and BP-ANN with SCG training and achieved overall accuracy of 98% for both of the classifiers. The Huang et al. [27] has built intelligent shoes for human identification under the framework of capturing and analyzing human gait. The data of that work were collected from different sensors, like a pressure sensor, a tilt angle sensor, three single-axis gyros, one tri-axial ACC and a bend sensor installed in the shoe. Principle component analysis (PCA) was used for feature generation, and SVM was applied for training and classifier generation. They were successful in achieving a 98% human identification rate. The Lugade et al. [28] used ANN to determine dynamic balance control, as defined by the interaction of the center of mass (CoM) with the base of support (BoS), during the gait in the elderly using clinical evaluation on gaits. Subjects were asked to walk at a self-selected comfortable speed across a 10 m walkway in that work. During ambulation, 29 retro reflective markers were placed on bony landmarks of the body, where 3D marker trajectories were captured with an eight-camera motion analysis system (Motion Analysis Corp, Santa Rosa, CA, USA). BP-ANN was able to correctly identify the interaction of the CoM with BoS of elderly subjects with an 89% accuracy rate.

The Muhammad et al. [29] used 25 reflective markers, which were placed on the body, and data were acquired from the Vicon Nexus 3D motion capture system to analyze the gait patterns, kinetic and kinematic parameters of the hip, knee and ankle joints of patients and normal subjects. They have used ANN to predict the gait patters with approximately 95% accuracy. The Ahlrichs et al. [30] used one tri-accelerometer device worn on the waist of the testing subjects used in detecting the freezing of gait (FOG) symptoms on the people suffering from Parkinson’s disease. The acceleration signals from a waist-mounted sensor are split into equally-sized windows (i.e., a sliding window is applied to the time series), and features are extracted from those windows and fed to an SVM for training or classification. The RBF kernel SVM achieved 98% accuracy in detecting the symptoms for the patients with Parkinson’s disease.

We have attempted to validate the measured WGAS data with the data taken from a video-based kinematic reference system; i.e., a Vicon motion capture system with cameras. However, so far, we have found it very difficult to validate the WGAS data directly this way, as the Vicon camera-based system measures the movements from the markers on the body, but not the exact force-based acceleration data, as measured from our WGAS. Therefore, a limitation of the current paper exists in the lack of a direct validation of the values of the extracted features to the measured data provided by another reference system (e.g., a kinematic system). However, we do plan to continue the next stage of the research by including this validation step against possibly another kinematic system and/or using a different validation method. In addition, to improve the data statistics of our work, we would like to add more data as we compared the performance of the classifiers with the increased normal data, as shown in Table 10 in the revised manuscript. The total dataset has four patients and twelve normal subjects, and the overall accuracies are still improved when compared with the results from Table 6.

## 4. Conclusions

The results presented in this work using our custom WGAS gait analysis system with artificial neural networks (ANN) and other classifiers, such as SVM, suggest that our low-cost system can successfully classify/detect patients with balance disorders from normal subject with very high accuracy and in almost no time. In this study, we used six simple features from our raw WGAS data collected during the DGI tests for seven subjects with BP-ANN SCG trained and/or linear SVM classifiers, and they achieved impressive 100%, 98% overall accuracies, respectively. In addition, KNN and binary decision tree (BDT) algorithms have obtained still pretty good 96% and 94% overall accuracies, respectively. We have also studied the performance of these classification algorithms when different input features (i.e., STDEV and range + STDEV) are used to train the classifiers, and the results are shown in Table 7 and Table 8, indicating that BP-ANN appears to have the best combined overall performance of classification accuracy and speed. We have also compared our gait classification results against prior works in the literature and confirmed that our work achieved very high classification accuracies, albeit with a limited subject size. We are in the process of collecting more data for patients of balance disorders at this moment. One of the goals of our study is to provide physicians and patients with a cost-effective means to identify dynamic balance issues and the possible risk of falls from data routinely collected in clinical examinations, such as the DGI tests reported in this work. In the future, we also plan to use our low-cost WGAS and classifiers, such as BP-ANN, to differentiate patients-specific gait issues to potentially form a powerful expert system, capable of real-time gait analysis to assist quantitative diagnosis, monitoring and assessment of fall risks and also to potentially help suggesting effective fall prevention schemes.

## Figures and Tables

**Table 1:** Dynamic Gait Index (DGI) tests performed for gait analysis.

Test/Case No.	Description
1	Gait level surface: walk with a normal speed up to the 20′ mark
2	Change in gait speed: walk with a normal pace up to 5′; walk fast for the next 5′; walk slowly for the next 5′; and walk normally for the last 5′
3	Gait with horizontal head turns: walk normally with horizontal head turns up to the 20′ mark
4	Gait with vertical head turns: walk normally with vertical head turns up to the 20′ mark
5	Gait and pivot turn: walk normally, but at the end, turn around with a pivot turn
6	Step over obstacle: walk normally, and when you come across the obstacle, step over, but not around it
7	Step around obstacles: walk normal, and when encountering the 1st obstacle, walk around the right-side; when encountering the 2nd obstacle, walk around the left-side

**Table 2:** The standard deviation (STDEV), range, mean, median and IQR (interquartile range) of DGI Test 2 data averaged for all normal subjects vs. patients.

STDEV-DGI Test 2						
Unit: in Voltage (V)	Gyro Z	Gyro X	Gyro Y	ACC Z	ACC X	ACC Y
Normal	0.0783	0.0202	0.0369	0.0051	0.0074	0.0076
Patients	0.0582	0.0570	0.0424	0.0375	0.0325	0.0270
Range-DGI Test 2						
Normal	0.4130	0.1513	0.1855	0.0302	0.0410	0.0312
Patients	0.5559	0.2230	0.2623	0.2112	0.1825	0.1861
Mean-DGI Test 2						
Normal	1.6048	2.2149	1.2785	2.9267	2.2080	2.2218
Patients	1.1907	1.8570	1.2795	2.0153	1.8551	2.4705
Median-DGI Test 2						
Normal	1.5926	2.2708	1.3076	2.9728	2.2325	2.2445
Patients	1.1869	1.8528	1.2813	2.0146	1.8561	2.4686
IQR-DGI Test 2						
Normal	0.0258	0.0976	0.0195	0.0293	0.0048	0.0053
Patients	0.0336	0.0484	0.0205	0.0255	0.0218	0.0260

**Table 3:** The STDEV, range, mean, median and IQR of DGI Test 7 data averaged for all normal subjects vs. patients.

STDEV-DGI Test 7						
Unit: in Voltage (V)	Gyro Z	Gyro X	Gyro Y	ACC Z	ACC X	ACC Y
Normal	0.1088	0.0606	0.0395	0.0158	0.0167	0.0197
Patients	0.0860	0.0592	0.0531	0.0280	0.0264	0.0244
Range-DGI Test 7						
Normal	0.7207	0.2011	0.2099	0.0683	0.0673	0.0820
Patients	0.4081	0.2228	0.2815	0.1678	0.1403	0.1311
Mean-DGI Test 7						
Normal	1.6300	2.2414	1.3458	2.9470	2.2183	2.2318
Patients	1.2041	1.8253	1.3104	1.9982	1.8384	2.2458
Median-DGI Test 7						
Normal	1.6359	2.2175	1.3447	2.9494	2.2208	2.2308
Patients	1.1937	1.8158	1.3104	1.9971	1.8380	2.4559
IQR-DGI Test 7						
Normal	0.0585	0.0551	0.0273	0.0131	0.0136	0.0156
Patients	0.0467	0.0043	0.0413	0.0141	0.0106	0.0121

**Table 4:** STDEV for three normal subjects and four patients for DGI Test 2.

STDEV of GYRO Z for DGI Test 2 (in Voltage)						
Normal 1	Normal 2	Normal 3	Patient1	Patient 2	Patient 3	Patient 4
0.0802	0.0746	0.0802	0.1021	0.0940	0.0155	0.0212

**Table 5:** STDEV, range, mean, median and IQR of all DGI Tests 1–7 data averaged for all normal subjects vs. patients.

STDEV-DGI Tests 1–7						
Unit: in Voltage (V)	Gyro Z	Gyro X	Gyro Y	ACC Z	ACC X	ACC Y
Normal	0.1033	0.0323	0.0372	0.0121	0.0138	0.0131
Patients	0.0809	0.0532	0.0468	0.0377	0.0382	0.0355
**Range-DGI Tests 1–7**						
Normal	0.625	0.1516	0.2292	0.0594	0.0739	0.0637
Patients	0.3854	0.2362	0.2649	0.2236	0.2004	0.1813
**Mean-DGI Tests 1–7**						
Normal	1.6262	2.2422	1.3278	2.9473	2.2137	2.2264
Patients	1.2200	1.8426	1.2884	1.9902	1.8345	2.4505
**Median-DGI Tests 1–7**						
Normal	1.6231	2.2456	1.3312	2.9554	2.2186	2.2308
Patients	1.2262	1.8415	1.2896	1.9890	1.8333	2.4492
**IQR-DGI Tests 1–7**						
Normal	0.0498	0.0226	0.0226	0.0057	0.0071	0.0071
Patients	0.0461	0.0391	0.0264	0.0224	0.0233	0.0222

**Table 6:** Comparison of classification algorithms using performance metrics when the range data are used as the input features.

Classifier	Sensitivity	Specificity	Precision	NPV	F-Measure	Accuracy
BP-ANN	100%	100%	100%	100%	1	100%
SVM	95.2%	100%	100%	96.5%	0.97	98%
KNN	95.2%	96.4%	95.2%	96.4%	0.95	96%
BDT	95.2%	92.8%	91%	96.3%	0.93	94%

**Table 7:** Overall classification accuracy of different algorithms using different input features.

Features	Overall Classification Accuracy (Patients vs. Normal Subjects)			
	ANN	SVM	KNN	BDT
Range	100%	98%	96%	94%
STDEV	92%	94%	89.8%	85.7%
Range + STDEV	96%	98%	94%	94%

**Table 8:** Summary of each classifier’s speed when different input features are used.

Features	Overall Classifier Speed (s)			
	ANN	SVM	KNN	BDT
Range	0.061	1.78	1.04	1.12
STDEV	0.052	1.92	1.88	2.06
Range + STDEV	0.97	2.80	1.76	2.31

**Table 9:** Our real**-**time WGAS gait classification system vs. the literature.

Reference	Algorithms (*)	Data Acquisition	Overall Accuracy
[22]	SVM	Three IMUs (Opal, APDM, Inc., Portland, OR, USA) featuring a tri-axial accelerometers and a tri-axial gyroscope	90.5%
[23]	ANN, SVM	37 markers were placed on body traced by an infrared camera during walking on the two embedded force plates	96.9%, 98.2%
[24]	ANN, SVM	2 markers on the shoe and traced by cameras during walking on a treadmill using the PEAK MOTUS motion analysis system	75%, 83.3%
[25]	ANN, SVM	Synchronized PEAK 3D motion analysis system and a force platform during normal walking	83.3%, 91.7%
[26]	ANN, SVM	Gait video sequence captured by a static camera during normal walking	98%, 98%
[27]	SVM with PCA	Integrated a pressure sensor, a tilt angle sensor, three single-axis gyroscopes, one tri-axial accelerometer and a bend sensor inside a small module in a shoe with an RF transmitter	98%
[28]	ANN	29 retro reflective markers placed on the body, with 3D marker trajectories captured with an 8-camera motion analysis system during normal walking	89%
[29]	ANN	25 reflective markers placed on the body and data acquired from the Vicon Nexus 3D motion capture system	95%
[30]	SVM	One tri-axial ACC worn on the subject waist while walking	98%
This work	ANN, SVM	tri-axial gyroscopes and tri-axial accelerometers integrated on a PCB forming a WGAS with an MSP430 microcontroller and an RF transmitter embedded on the PCB	100%, 98%

* SVM: support vector machine; ANN: artificial neural network; PCA: principle component analysis.

**Table 10:** Comparison of the classification algorithms using performance metrics when the range data are used as the input features (4 patients and 12 normal subjects).

Classifier	Sensitivity	Specificity	Accuracy
BP-ANN	100%	100%	100%
SVM	98.80%	96.30%	98.21%
KNN	96.42%	96.42%	96.42%
BDT	96.42%	89.28%	94.60%
